# A Novel Approach for Automatic Detection of Driver Fatigue Using EEG Signals Based on Graph Convolutional Networks

**DOI:** 10.3390/s24020364

**Published:** 2024-01-07

**Authors:** Sevda Zafarmandi Ardabili, Soufia Bahmani, Lida Zare Lahijan, Nastaran Khaleghi, Sobhan Sheykhivand, Sebelan Danishvar

**Affiliations:** 1 Electrical and Computer Engineering Department, Southern Methodist University, Dallas, TX 75205, USA; 2 Department of Computer Engineering and Information Technology, Amirkabir University of Technology, Tehran 15875-4413, Iran; 3 Biomedical Engineering Department, Faculty of Electrical and Computer Engineering, University of Tabriz, Tabriz 51666-16471, Iran; 4 Department of Biomedical Engineering, University of Bonab, Bonab 55517-61167, Iran; s.sheykhivand@tabrizu.ac.ir; 5 College of Engineering, Design and Physical Sciences, Brunel University London, Uxbridge UB8 3PH, UK

**Keywords:** deep learning, EEG, fatigue, GAN, GCN

## Abstract

Nowadays, the automatic detection of driver fatigue has become one of the important measures to prevent traffic accidents. For this purpose, a lot of research has been conducted in this field in recent years. However, the diagnosis of fatigue in recent research is binary and has no operational capability. This research presents a multi-class driver fatigue detection system based on electroencephalography (EEG) signals using deep learning networks. In the proposed system, a standard driving simulator has been designed, and a database has been collected based on the recording of EEG signals from 20 participants in five different classes of fatigue. In addition to self-report questionnaires, changes in physiological patterns are used to confirm the various stages of weariness in the suggested model. To pre-process and process the signal, a combination of generative adversarial networks (GAN) and graph convolutional networks (GCN) has been used. The proposed deep model includes five convolutional graph layers, one dense layer, and one fully connected layer. The accuracy obtained for the proposed model is 99%, 97%, 96%, and 91%, respectively, for the four different considered practical cases. The proposed model is compared to one developed through recent methods and research and has a promising performance.

## 1. Introduction 

Millions of people die in traffic accidents every year. Among driving accidents, fatigue and sleepiness are the main and major contributors to the occurrence of injuries and financial losses for drivers and passengers [1,2]. According to the statistics of the World Health Organization, 20 to 30 percent of road accidents are due to driver fatigue [3]. Based on the above facts, it is necessary to design an intelligent system to monitor driver fatigue and inform the driver of the degree of fatigue. Accordingly, in order to minimize injuries and prevent accidents, automatic fatigue-detection systems have been introduced as a way to ensure the safety of drivers and are currently available in several brands of the automotive industry [4]. These systems include monitoring and assessing the driver’s condition and can also include automatic alerts if any drowsiness is detected. It has been proven that 60% of accidents can be prevented by using warning systems with a warning time of half a second before an accident [5].

Driver fatigue can be divided into sleep-related (SR) and work-related (TR) fatigue based on causal factors [6]. SR fatigue is mainly influenced by circadian rhythms and sleep deprivation and restriction. TR fatigue is caused by driving tasks and tasks such as focusing on the road, turning the steering wheel, keeping the neck up, shifting gears, and using the pedals. This fatigue can be divided into two states: active fatigue and passive fatigue [7]. Active fatigue generally refers to overly mental driving conditions. A real-world example of this type of fatigue can be high traffic or the need for a secondary task while driving, such as finding an address [4]. On the other hand, the passive fatigue experienced by conditions such as road uniformity and low traffic is felt by the driver. In this scenario, the driver mainly monitors the driving environment; that is, driving becomes a predictable thing for the driver, which will reduce effort and skill and ultimately cause drowsiness in the driver. The current research also focuses on the same type of fatigue, i.e., passive TR fatigue.

As mentioned in the first paragraph, in recent years, various monitoring systems have been provided to monitor driver fatigue. In general, these systems are divided into the following three categories: monitoring based on image processing [8], monitoring based on vehicle indicators [9], and monitoring based on physiological indicators [10]. In monitoring systems based on image processing, a camera is placed in front of the drivers and it monitors the driver’s facial condition, including blinks and facial expressions. Despite the good performance, these systems are weak when the driver wears sunglasses. Also, these systems are highly dependent on the lighting conditions of the car cabin. Monitoring based on vehicle indicators is also possible by placing sensors on the steering wheel and placing cameras on the four sides of the vehicle. These systems can report the level of fatigue to the driver by monitoring the condition of the road markings and steering wheel movements. However, these systems are highly sensitive to weather and atmospheric conditions. Compared to driver fatigue-monitoring systems, monitoring based on physiological indicators can be considered the most reliable driver fatigue-monitoring system. These monitoring systems, which are based on the measurement of EEG, electromyography (EMG), electrocardiography (ECG), and electrooculography (EOG) signals, can detect acute fatigue in drivers before it occurs and notify the driver. Among the signals utilized to detect driver fatigue in these systems, it has been proved that EEG signals are the most accurate indicators for recognizing and diagnosing driver fatigue; this is because EEG changes its mode before the appearance of sleepiness in drivers and departs its regular pattern. Based on this, it is possible to inform the driver before the accidents occur and prevent the occurrence of driving accidents. The proposed system in this study is also designed based on monitoring that is based on physiological indicators, which, in turn, are based on recording EEG signals.

The main problem in monitoring the driver’s fatigue using EEG signals is as follows [11]:

First, previous studies have considered driver fatigue monitoring only in binary form and in two states of fatigue and normalcy. The use of binary driver fatigue-monitoring systems makes it impossible to implement research in the real world. Second, most of the algorithms used in previous studies are organized based on feature selection/extraction in an engineering and manual way. Manual approaches in driver fatigue studies cannot ensure the optimality of the feature vector for classification and cannot be applied in real-time applications. Thirdly, the number of EEG channels used in previous studies is large, and this can cause computational complexity and disturb drivers in real-time applications. Fourth, some recent studies have used deep learning networks for feature selection/extraction and classification. However, their classification accuracy is below 90%. Fifth, previous studies have used self-report questionnaires to confirm driver fatigue, and in case of incorrect self-reports from drivers, the algorithm will be associated with an error.

In order to solve the challenges of previous studies, the present study presents a new method for the automatic multi-level detection of driver fatigue using EEG signals. Accordingly, in the proposed method, a comprehensive benchmark database will be registered for the automatic detection of driver fatigue based on the minimum channel of EEG signals. After data augmentation using generative adversarial networks (GANs) from deep graph convolutional networks (GCNs) that have good resistance to environmental noises, including sounds related to the car engine, cabin, and passenger voices, in order to make a selection, end-to-end feature extraction and classification will be used. The confirmation of driver fatigue in this research will be performed based on the physiological changes of ECG, EMG, and respiratory effort and will not be limited to self-report questionnaires as in previous studies.

The contributions of this paper can be marked as the following:

(i) Providing a fully automatic system (end-to-end) to monitor driver fatigue by combining GAN-GCN networks.
(ii) Automatic detection of driver fatigue in five separate classes and four practical cases for the first time relative to previous research.
(iii) Building a driving simulator and collecting a comprehensive EEG database.
(iv) Achieving the highest performance for the classification of different classes of fatigue compared to previous research.

The remaining parts of this article are ordered as follows. The second part reviews recent studies to monitor driver fatigue one by one. The third part describes the theoretical performance of the algorithms used in this research, including GAN and GCN. The third part examines how to build a driving simulator and how to record the signal along with the proposed deep architecture. The fourth part is related to the obtained results, and finally, the fifth part is related to the conclusion.

## 2. Related Works

In this section, recent studies related to the diagnosis of driver fatigue are highlighted. In the first part, the research that is based on the traditional methods of feature selection/extraction in machine learning will be reviewed, and in the second part, the methods that are based on approaches related to deep learning networks will be discussed.

### 2.1. Related Methods Based on Manual Feature Selection/Extraction

Ren et al. [12] presented a new hybrid system for the fatigue detection of ship operators based on EEG signals. These researchers have used principal component analysis (PCA) to reduce the dimensions of EEG channels. Also, they used the features of center frequency and power spectral entropy (PSE) from channels 7 and 10 of EEG signals. The final performance of the model of these researchers was very promising. However, one of the weak points of this research is that it is sufficient to confirm fatigue based on the self-report questionnaire of the fatigue severity scale (FSS). Fouad et al. [13] presented a new model to monitor two levels of driver fatigue. These researchers used the EEG signals of 12 subjects in their study. They used a Support Vector Machine (SVM) and K-Nearest Neighbor (KNN), Random Forest (RF), and Bayesian classifiers for feature vector classification. The use of three channels of EEG signals was one of the merits of this research. Kumar et al. [14] used five different machine learning approaches to automatically detect driver fatigue. The approaches used in this research included multidimensional scaling (MDS), singular value decomposition (SVD), three-phase mean-based support vector regression, adjustable q-wavelet transform, and extreme learning machine (ELM). Also, in this research, they used classifiers such as Adaboost and SVM to classify the feature matrix. Finally, these researchers achieved a high accuracy of 99% for the classification of two classes, normal and fatigue. Lee et al. [15] presented a statistical model using fatigue evaluation criteria, including the Epworth sleepiness scale and Carolina sleepiness scale, in order to evaluate driver fatigue. In this research, they used 32 channels of EEG signals. In their study, these researchers collected an EEG database of 63 train drivers and 30 car drivers. The study of these researchers showed that theta, alpha, and beta waves have major changes during fatigue. Ha et al. [16] presented a new model using EEG signals to automatically detect driver fatigue. These researchers used EEG signals of 48 car drivers in their study. Their proposed model was composed of a combination of fuzzy delay index and SVM. The accuracy obtained by these researchers is reported to be above 93%. Among the advantages of this research was the lack of computational complexity in the proposed model.

### 2.2. Related Methods Based on Deep Learning Networks

In the following, recent research that is based on deep learning is reviewed.

Abdubrani et al. [17] presented a new model for automatic driver fatigue detection. These researchers used 12 participants and recorded the EEG signals of the participants. After recording the signal, the PCA algorithm was used to identify the active channels. The channels selected by these researchers included O1, O2, FP1, FP2, P3, P4, F3, and F4. Feature extraction in this research was performed using continuous wavelet transform (WT), and classification was performed based on four different networks, including recurrent neural networks (RNN), deep convolutional networks, artificial neural networks (ANN), and SVM. Among the networks used for classification, ANN showed the best performance. The accuracy obtained in this work for the binary classification of driver fatigue was higher than 95%. Among the disadvantages of this research was the high computational volume, which is not recommended for real-time applications. Sheykhivand et al. [18] used deep learning approaches to automatically detect two levels of driver fatigue. In their research, these researchers evaluated several areas of the EEG signals to detect driver fatigue. Their architecture consisted of a combination of CNN and long short-term memory (LSTM) networks. These researchers achieved a high accuracy of 99% in order to classify the two classes of normal and fatigue. The advantage of this research was the high classification accuracy, and the computational complexity can be considered as a limitation of this study. Sheykhivand et al. [19] combined compressed sensing (CS) and deep learning approaches to automatically detect driver fatigue. These researchers first compressed the EEG signals recorded from 12 participants and then directly entered the compressed matrix into the CNN. The obtained results showed that this research had a promising performance for real-time applications. The limitation of this study can be considered in the number of binary states for classification. Peivandi et al. [20] used physiological signals to automatically detect driver fatigue. In their research, these researchers used EEG, EMG, ECG, and EOG signals to automatically detect driver fatigue. The network architecture of these researchers included the combination of CNN and type-2 fuzzy networks. In this study, type-2 fuzzy functions were used instead of Relu and Leaky Relu activation functions to avoid various uncertainties. The accuracy of multi-level classification in this study is reported to be above 89%. Gao et al. [21] presented a new model based on a logarithmic spectrogram and convolutional-RNN based on EEG signals. The proposed architecture of these researchers included six convolutional layers with a two-way RNN. In their model, spectrogram features were transferred to the convolutional network to extract high-level features from it. Then, temporal features are extracted by recurrent networks. Finally, the feature matrix was classified into two classes of fatigued and normal states by a fully connected classifier, a Relu activation layer, and a softmax function. The performance of this research was compared with four different approaches, and it was able to show the best performance. Chen et al. [22] used deep learning approaches to automatically detect driver fatigue. These researchers used a capsule network connected to the self-attention channel (CapsNet-SACC) for the feature selection/extraction and classification of two different states of tiredness and normality. In this study, they used the EEG data of 21 participants. These researchers have come to the conclusion that the frontal areas of the head can be effective in detecting driver fatigue. Recent studies reviewed are summarized in Table 1.

## 3. Materials and Methods

The theoretical foundations of the algorithms utilized in this study are addressed in this part.

### 3.1. Brief of Generative Adversarial Network (GAN)

The need to train deep learning networks with a small amount of data has been the subject of studies that have attracted the attention of researchers in recent years. In years past, classical methods, including the overlap technique, have been used to increase the database. However, the use of this technique leads to the artificial expansion of the data, which makes the accuracy of the classification ambiguous. With the expansion of deep learning networks, GANs were presented to increase the volume of data sets [23,24].

### 3.2. Brief of Graph Convolutional Network (GCN)

The basic idea of the GCN was first proposed by Michael Deferard et al. in 2016. For the first time, these researchers have used signal processing in the field of graphs and graph spectral theory in the definition of convolutional function, which enables the use of convolutional networks in the form of graph theory.

In graph theory, having a degree matrix and an adjacency matrix is of special importance. The connection of each vertex in the graph is achieved using the adjacency matrix. Also, by having the adjacency matrix, the degree matrix can also be obtained. This matrix is a diagonal matrix, in which the matrix diagonal elements are equal to the sum of the edges connected to the vertex corresponding to the matrix [25]. 

## 4. Proposed Model

**Figure 1:**
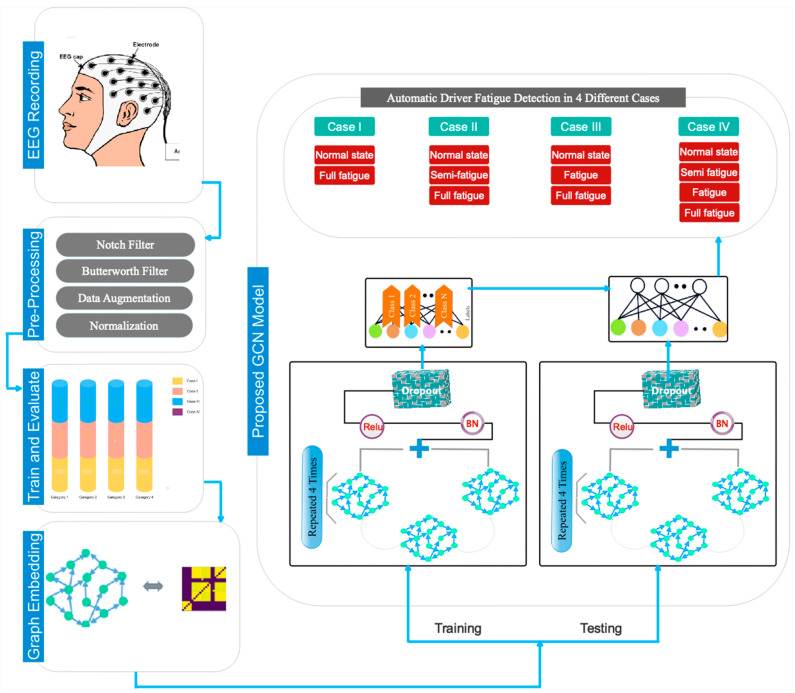
The proposed GCN model for automatic classification of driver fatigue in 4 application cases.

In this section, the method of registering the database and the proposed model of the current research will be presented. Figure 1 shows the organizational structure and proposed GCN paradigm. 

### 4.1. Collected Database

In this section, details related to database collection will be provided. This section includes three sub-sections of driving simulator design, the collection of physiological signals, and considered trims.

#### 4.1.1. Driving Simulator Design

**Figure 2:**
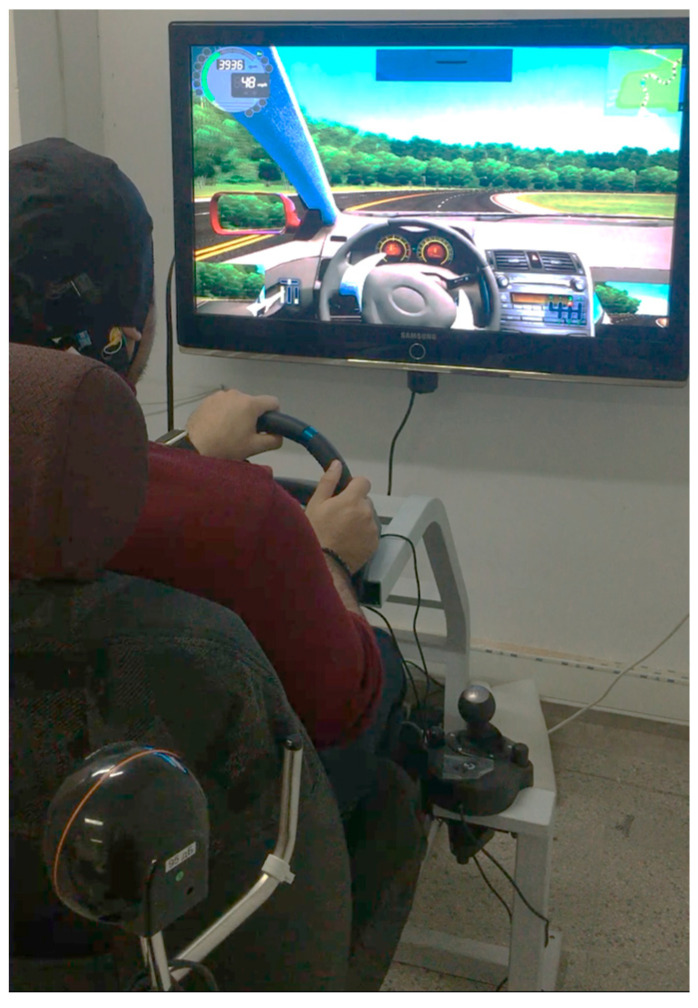
Recommended designed driving simulator.

To monitor driver fatigue, it was necessary to design and build a driving simulator. To achieve this goal, a cost-effective and standard simulator was designed and built in the signal processing laboratory of the University of Tabriz. For this purpose, the steering wheel, automatic transmission, and gas and brake pedals of the company Logitech were used in the design of the simulator. Also, in the proposed simulator, a 50-inch Samsung screen was used instead of the windshield of the car. The front chest of the car, which holds the steering wheel, was designed and built in the proposed simulator using a metal profile with adjustable dimensions for participants with different heights and weights in Corel software ver 2.4. In order to avoid distracting the participants, separating panels were used for the car cabin so that the participants could only focus on the screen in front of them while driving. An adjustable car seat was also used in the simulator. Figure 2 shows the designed simulator.

In almost none of the simulators designed in the previous studies, the sound of the engine and car cabin is not considered in order to lower the classification accuracy. In order to find the way to the practical field of the present research to automatically detect driver fatigue, these uncertainties must be fully applied in the testing process. Accordingly, in order to induce the sound of the car’s cabin and engine, four stereo speakers were used in the simulator. The loudness of the engine and car cabin was measured in real mode on a highway using sound meter software ver 6, and the same volume was set in the simulator. The premium version of Car City Driver simulator software was used in the designed driving simulator. The driving situation in the software was set to day mode, automatic transmission mode, and on a highway without traffic to create a state of mental fatigue in the people participating in the simulator.

#### 4.1.2. Collection of Physiological Signals

**Figure 3:**
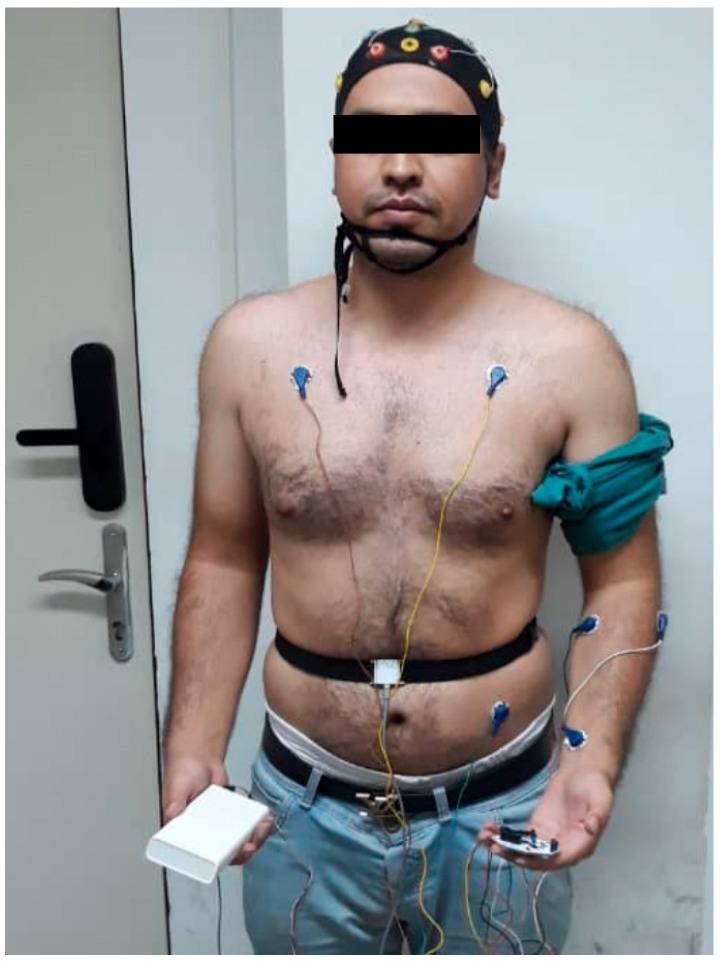
Connecting the cap, electrodes, and sensors to measure physiological signals in one of the participants.

A total of 20 bachelor’s, master’s, and doctoral students (10 women and 10 men) aged between 20 and 35 years without any history of illness participated in the driver fatigue-monitoring test. Informed consent was obtained from all participants in the experiment and approved by the ethics committee of Tabriz University with the license number IR.Tabrizu.1399.6.1. All the participants in the test had a driver’s license and had no previous driving experience in the simulator. Then, 24 h before the experiment, the participants were asked to refrain from taking any kind of medicine, energy drinks, or caffeine. Also, all participants were asked to take a bath before the experiment and refrain from using hair conditioners. The recording of EEG signals in this experiment was performed by the Russian Encephalan device. The available device had 21 electrodes with a sampling frequency of 250 Hz. In this device, electrodes A1 and A2 were related to the reference, and the other 19 electrodes were used to record the EEG signals. Electrode placement and signal recording were performed based on the 10–20 system, and the resistance between the electrode and the scalp was set below 5 kΩ. In the recording of EEG signals, three different gels of American, Russian, and German brands were used. The gel used from the American brand had the best performance in terms of setting the resistance below 5 kΩ between the electrode and the skin. As mentioned in the review section of previous research, previous research evaluated the fatigue of drivers based on self-report questionnaires. The use of these questionnaires is unprincipled and can cause a disturbance in the correct diagnosis because of incorrect self-statements from the drivers. To solve this problem, in addition to using the questionnaire, we have also recorded other physiological signals, including ECG, EMG, and respiratory effort signals. These signals are recorded along with the EEG signals from the participants in the experiment. However, in the process of processing using the proposed architecture, only EEG signals have been used, and other physiological signals have been intuitively and qualitatively examined by a neurologist just to confirm fatigue. To achieve this goal, the OpenBCI module was used synchronously with the Encephalon device in order to record physiological signals. To record the ECG signal, three leads with sticky electrodes (two electrodes for recording and one electrode for reference) were used in the chest of the participants with a sampling frequency of 500 Hz. Also, EMG signals were recorded using three sticky electrodes (two electrodes for recording and one electrode for reference) with the same frequency on the wrists to control steering wheel movements. In addition, a sensor belt was used to monitor participants’ inhalation and exhalation to determine the drivers’ breathing efforts during the test. How to connect the cap, electrodes, and sensors to measure physiological signals is shown in Figure 3.

#### 4.1.3. Considered Trims

**Figure 4:**
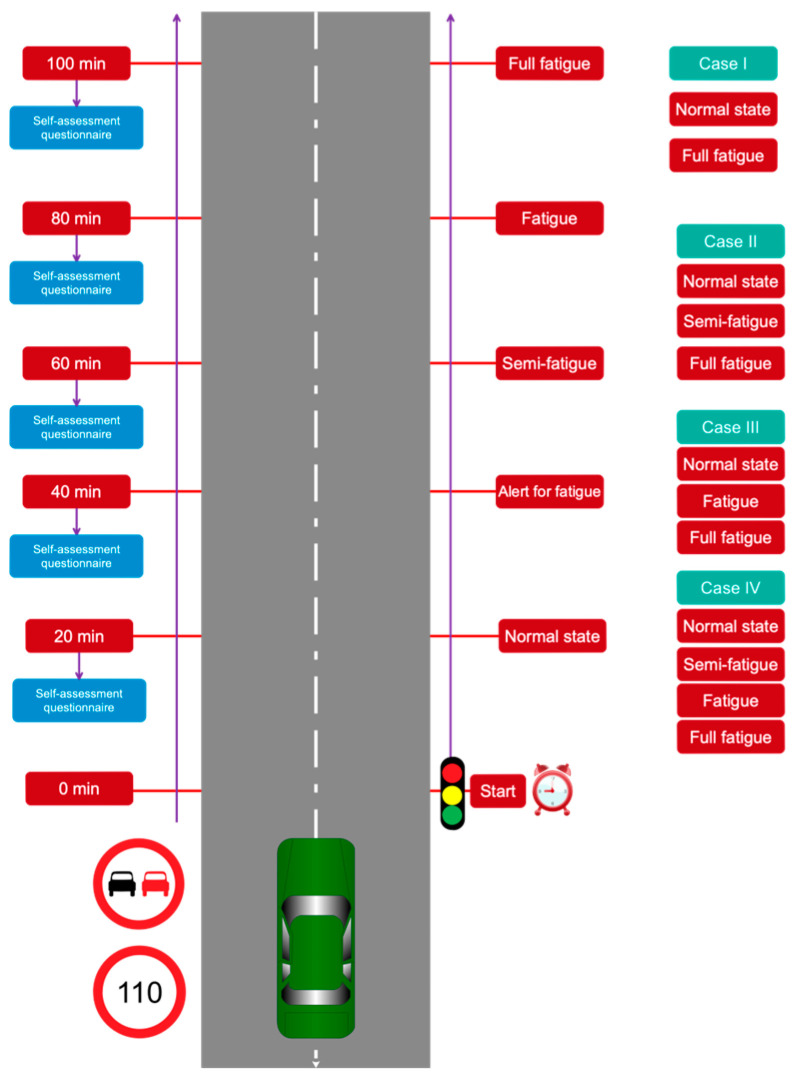
Trims intended for the participants to drive along with class stickers and utility cases.

All the tests were performed at a certain time in the morning so that the participants would not feel tired from the daily work. Before starting the test, the objectives of the experiment were explained to the participants. Also, the participants practiced with the simulator for 3 min to familiarize themselves with the driving simulator and its environment. We have considered five different stages in four separate modes for driver fatigue monitoring, which is graphically displayed in Figure 4. According to this figure, at first, the drivers drove for 20 min, during which all the physiological signals were obtained from the participants. Then, the participants completed the Chalder Fatigue Self-Report Questionnaire [26,27,28]. The previous step was repeated four more times for the participants in the experiment, and the experiment ended. The EEG signals recorded in the five mentioned stages were labeled in five different classes with the names normal, alert for fatigue, semi-fatigue, fatigue, and full fatigue, respectively. In this research, we have analyzed, evaluated, and classified five registered classes in four different cases, according to Figure 4.

### 4.2. Pre-Processing of the Proposed Model

As we know, EEG signals are very sensitive to noise, which must be minimized by techniques and algorithms before entering the proposed architecture. Accordingly, pre-processing was performed after recording the signal, which will be reviewed below.

It is generally known that mental fatigue can alter EEG measurements in a discernible way. When the EEG is resting, theta and alpha waves exhibit significant positive and negative associations, respectively, with subjective weariness. As mental fatigue develops, the alpha rhythm’s intensity rises when the eyes are open and falls when they are closed [16]. Based on the above information, out of 19 electrodes related to EEG, only 4 electrodes, including P4, C3, O1, and O2, have been used to detect driver fatigue automatically [16,17]. Accordingly, the rest of the electrodes are not included in the processing process. Also, considering that one of the innovations of this research is the use of the minimum window of EEG signals to detect fatigue, in each class, only 5 s of the signal (from the end of each recorded signal) is used for detection, and the rest of the recorded signals are excluded from the processing process.

A Notch filter (Second-Order Bandstop) [29] was applied to the recorded signals in order to remove the 50 Hz frequency of city electricity. Then, the recorded signal was filtered by a second-order Butterworth filter [30] with a frequency of 0.02 to 50 Hz. In order to avoid the phenomenon of overfitting, GAN networks (described in Section 2) were used to increase the recorded data. For this purpose, a computationally efficient trial-and-error architecture was chosen for the generative and discriminating subnets. Based on this, the generative network with four convolutional layers along with the Relu activation function with dimensions of 128, 256, 512, and 1250 in each layer was selected. The discriminator network also had four fully connected layers. There was also a random elimination layer in the first layer of the discriminant network. The learning rate in the proposed GAN network in this study was determined to be 0.001, and the number of 500 iterations was set for the network. Also, Adam’s optimizer has been used to optimize parameters in this network. Finally, the number of samples increased from 1250 to 2920. After increasing the number of samples, the generated data were normalized between 0 and 1 using a Min-Max normalizer [31] in order to improve the training process in the data processing stage. After the aforementioned pre-processing, the data were ready for the processing stage in the next section.

### 4.3. Proposed GCN Model

In this section, the proposed network architecture will be fully explained along with relevant details, including the number of layers considered and the type of layers used.

#### 4.3.1. Graph Formation

An adjacency matrix is generated by calculating the functional connectivity between channels in the EEG data. This is performed by measuring the correlation between channels and representing it as a matrix called the connectivity matrix of the EEG channels. To derive the adjacency matrix of the network, a threshold is set to sparsely approximate the connectivity matrix. The generated graph is inputted into the proposed GCN, which includes feature selection/extraction and classification phases (end-to-end).

#### 4.3.2. Designed Architecture

**Figure 5:**
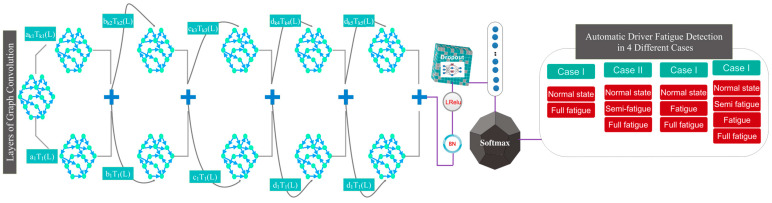
Architecture designed based on graph convolutional network.

**Figure 6:**
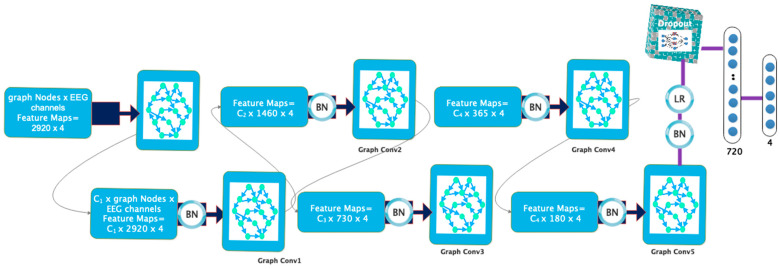
The dimensions of the layers of the proposed GCN model.

The architecture of the proposed model consists of a number of convolutional graph layers that perform feature selection/extraction and hierarchical (end-to-end) classification from the recorded data. Also, graph convolution layers extract dynamic information from the connected EEG channels. The architecture of the model presented in this research uses five convolution graph layers. Graph approximation in each convolution graph layer is performed using Chebyshev polynomial expansion. In each of the layers of the convolution graph, the Leaky Relu activation unit is used. Also, the output of each layer is connected to batch normalization to speed up the model-training process and make the network more stable. The feature vector extracted from the fifth layer passes through a dropout layer to avoid overfitting the model during the training process. A flattening layer is then used before labeling. Finally, the output of the previous step reaches the fully connected layer along with a softmax function to perform the labeling operation in order to automatically detect the driver’s fatigue in different cases. The operations described in this section are shown graphically in Figure 5. Also, Figure 6 specifies the size of the dimensions in the layers in the proposed model. According to Figure 6, the reduction of dimensions in the layers is clear. These dimensions are determined based on the sampling frequency and the duration considered in each fatigue stage. In the proposed architecture, the number of EEG channels considered is equal to the number of nodes in the GCN model. Accordingly, in the first layer of convolution, each node in the constructed graph will have 2920 samples per vertex. The weight dimensions, along with the details of the layers, including the number of parameters, are shown in Table 2. As can be seen, coefficients C1, C2, C3, and C4, which show the expansion of Chebyshev polynomials in each layer of the convolution graph, are different in each layer.

### 4.4. Training and Evaluation of the Proposed GCN Model

The training and evaluation of the proposed GCN model is chosen by trial and error. Also, the weights in the proposed model were initially set very small and were optimized by Adamax’s algorithm in each iteration. The error function in the cross-entropy network is considered. All the trials and errors made in order to reach the best architecture are summarized in Table 3.

As mentioned earlier, the total number of samples for each class was 2920, of which 70% were selected for the training set, 20% for the validation set, and 10% for the test set. This method of data allocation is based on the following recent studies related to the field of driver fatigue diagnosis.

## 5. Results and Discussion 

This section includes four sub-sections, including the optimization results of the proposed architecture, the results obtained from the proposed GCN model, a comparison with recent research and algorithms, and a discussion on fatigue verification.

We have used the premium version of Google Colab to present simulation results. Also, we have used Matlab software ver 2020 to present data pre-processing results and Python programming language to design the proposed GCN model.

In this research, the criteria of accuracy, precision, and sensitivity have been used to evaluate the proposed model. The equation of evaluation criteria is presented as follows:

(1) $$Accuracy=\frac{TP+TN}{TP+TN+FP+FN}$$

(2) $$Precision=\frac{TP}{TP+FP}$$

(3) $$Sensitivity=\frac{TP}{TP+FN}$$

According to the above equations, true positive (*TN*), true negative (*TN*), false positive (*FP*), and false negative (*FN*) samples are in classification classes.

### 5.1. Optimization Results of the Proposed Architecture

**Figure 7:**
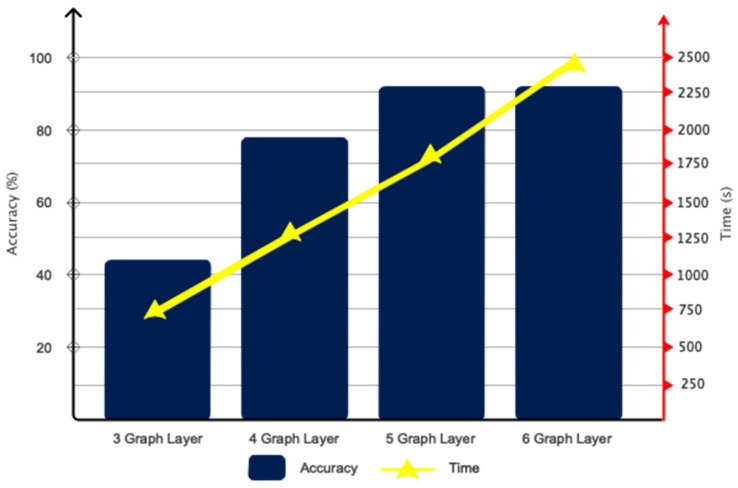
Considered graph convolutional layers along with training time.

**Figure 8:**
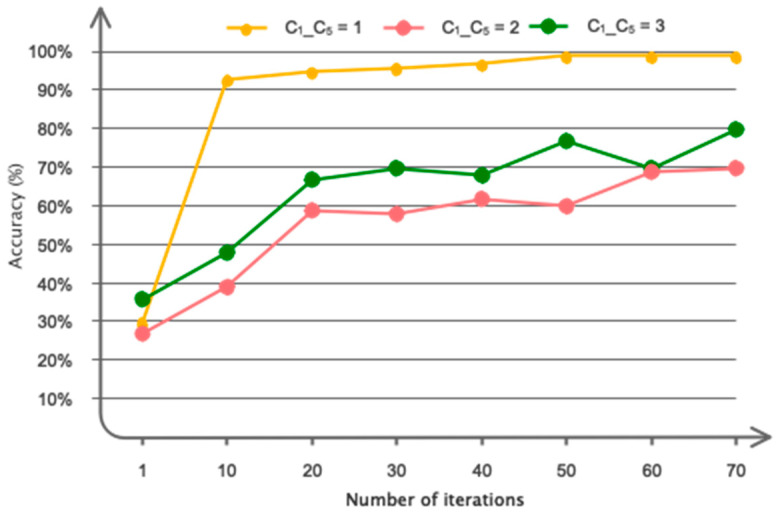
Different considered dimensions for coefficients of Chebyshev polynomial expansion.

As explained in the architectural design section, the GCN model is developed based on trial and error. The results of the number of considered layers, along with the duration of training for automatic driver fatigue detection, are presented in Figure 7. According to this figure, as can be seen, a number of five convolutional graph layers optimized accuracy and time. According to the figure, if considering six layers, accuracy does not change much. However, the duration of training increases. Accordingly, the number of layers in the proposed GCN model is five. Various coefficients were considered in the proposed architecture for Chebyshev polynomial expansion. The results obtained for the considered coefficients are shown in Figure 8. Accordingly, as it is known, attributing the number 1 for the coefficients of C_1_–C_5_ has maximized the accuracy of the classification.

### 5.2. The Results Obtained from the Proposed GCN Model

**Figure 9:**
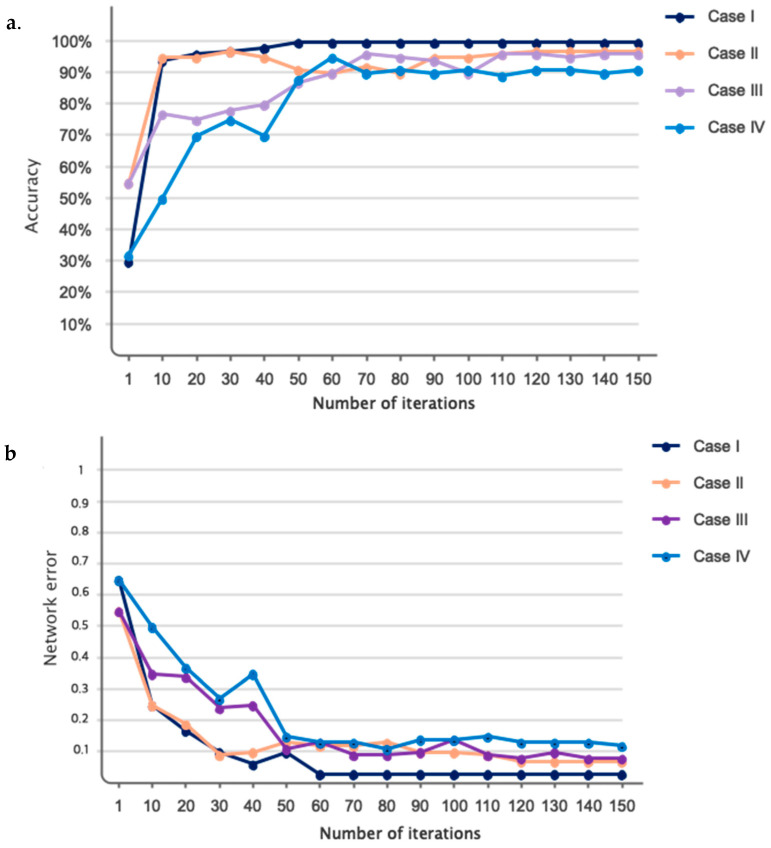
Performance of the proposed GCN model in terms of (**a**). classification accuracy and (**b**). error.

**Figure 10:**
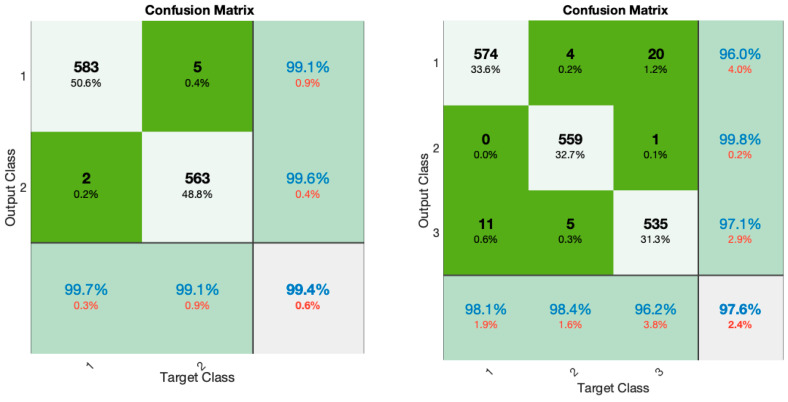

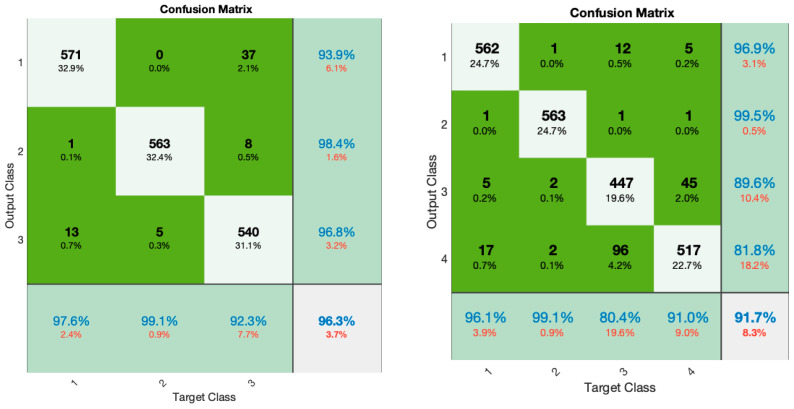
Confusion matrix is considered for four cases.

**Figure 11:**
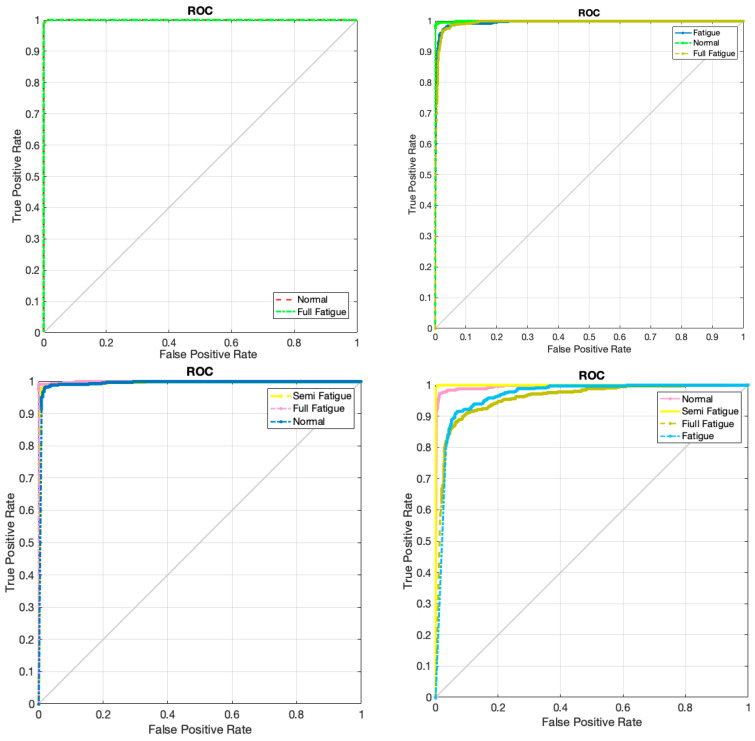
ROC diagram for four different cases considered in order to automatically classify driver fatigue.

**Figure 12:**
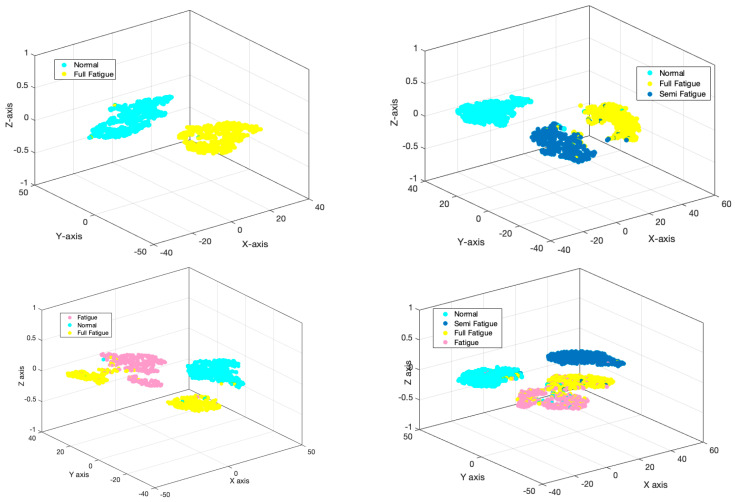
Scatter plot of samples for fully connected layer in GCN model.

The accuracy and error of the proposed GCN model for the classification of four different cases (checked in Figure 4) are shown in Figure 9 for 150 iterations. As can be seen from Figure 9a, the classification accuracy for case I, which is the classification of full fatigue and normal, is around 99.1%. Also, according to this figure, as clearly shown, the accuracy of the classification for all cases considered for the automatic detection of driver fatigue is above 90%, which shows the acceptable performance of the designed architecture. Figure 9b shows the error of the proposed model for all considered cases. According to this figure, as seen by increasing the number of GCN model iterations, the number of errors has decreased, so for case I, the error increased from 0.65 to 0.01. Figure 10 shows the confusion matrix for the four considered cases of two classes, three classes, and four classes. As can be seen, only two samples for case I are misdiagnosed as the fatigue state. Figure 11 shows the Receiver Operating Characteristic (ROC) for the considered cases. As it is known, for all the considered cases, the left quadrant is in the range of 0.9 to 1, which shows the optimal performance of the proposed architecture. Figure 12 shows the scatter plot of the samples of each case for the FC layer. Based on the T-SNE diagram, as it is known, almost all the samples are separated from each other in the FC layer.

Table 4 examines the performance of the GCN model in terms of various evaluation criteria, including sensitivity and kappa coefficient. According to this table, as clearly shown, the proposed model has been able to perform significantly for different considered cases.

### 5.3. Comparison with Recent Algorithms

**Figure 13:**
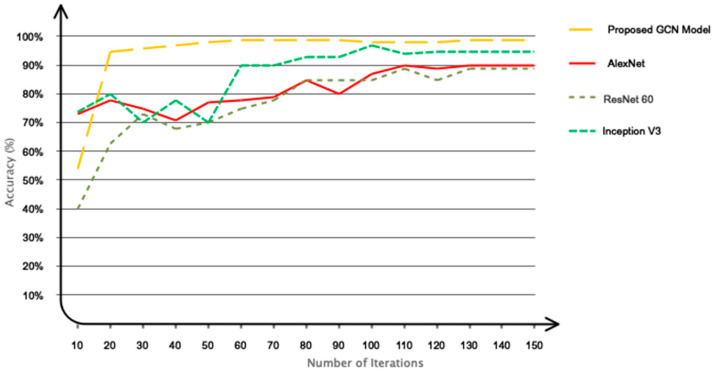
Performance of the proposed model against pre-trained networks.

We have evaluated the GCN model with commonly used algorithms in previous studies (including Alex Net, ResNet 60, and Inception V3) based on our own collected database. The obtained results are shown in Figure 13. As can be seen, the proposed GCN model has higher convergence speed and more accuracy compared to AlexNet and Inception networks.

**Figure 14:**
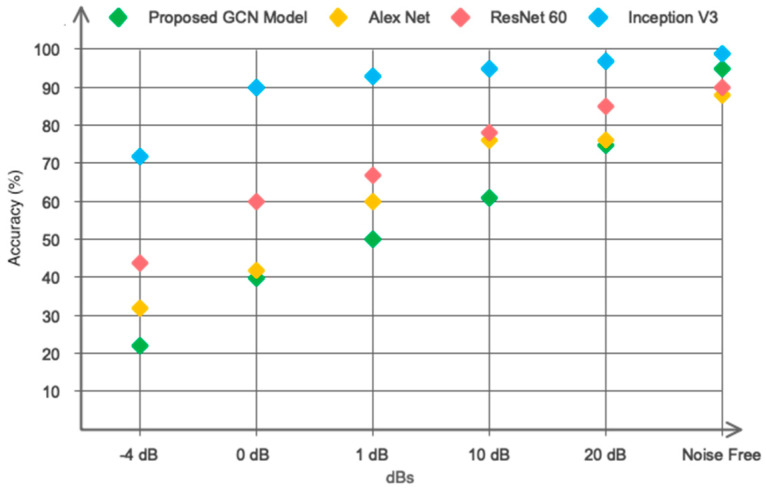
Performance of the proposed model in noisy environments.

As mentioned in the database collection section, none of the recent studies have considered engine and cabin noise in their study in order to avoid reducing the classification accuracy. We have considered the recorded signals along with the sound of the car’s cabin and engine. In addition, we artificially added a Gaussian white noise in a large range of different SNRs to the recorded data to measure the robustness of the GCN model with the recently used algorithms. The obtained results are presented in Figure 14. As can be seen, the classification accuracy of the proposed GCN model remains above 90% until SNR = 0 dB.

### 5.4. Discussion on Fatigue Verification

**Figure 15:**
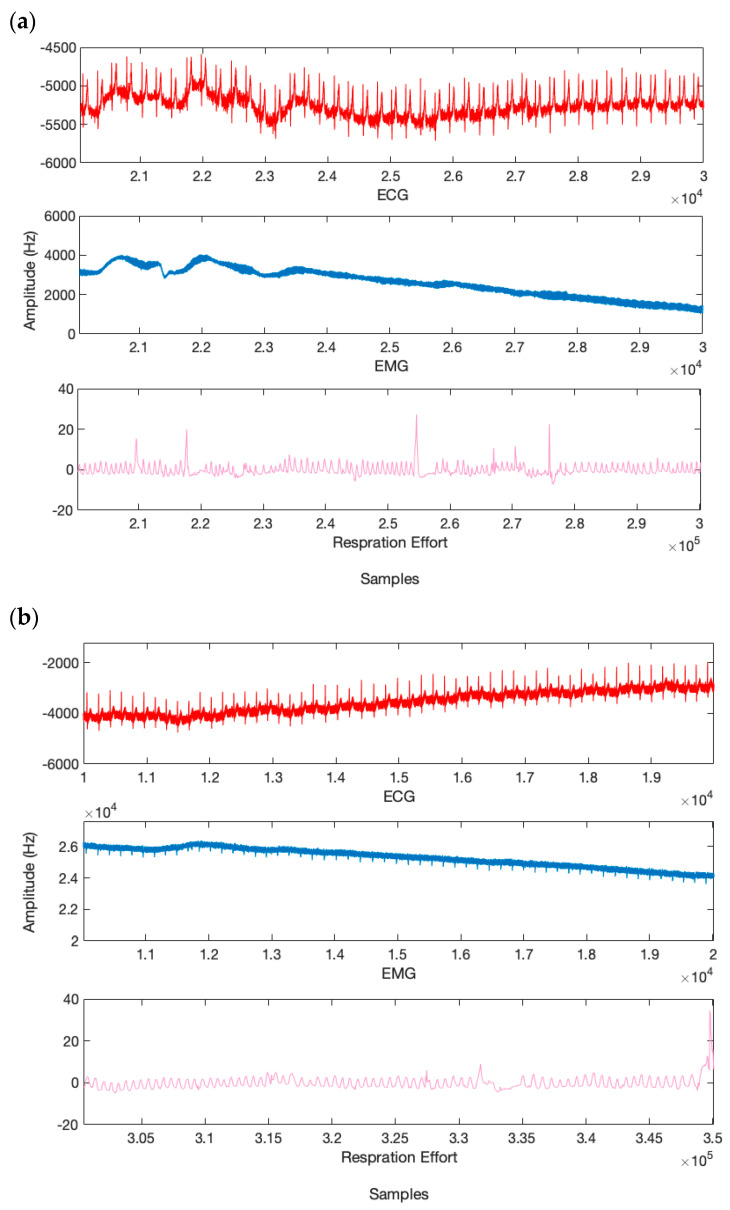
Comparison of physiological signals in normal and complete fatigue. (**a**) normal stage, (**b**) fatigue stage.

Fatigue has been confirmed in previous studies based on self-report questionnaires. Relying on a questionnaire cannot be a reliable method to confirm drivers’ fatigue because drivers’ statements can be dishonest. For this reason, as described in the database registration section, in our study, in addition to recording EEG signals, we have recorded ECG, EMG, and respiratory effort signals from the participants. A neurologist confirmed the fatigue of each of the participants based on the physiological signals, so that the fatigue is not confirmed solely based on self-report based on the questionnaire. The recorded physiological signals are shown in Figure 15. Figure 15a,b show the recorded signals for normal and full fatigue, respectively. Based on Figure 15b, as can be seen, the number of heartbeats has decreased, which indicates the fatigue of the driver. Also, during complete fatigue, the degree of freedom of steering by the driver decreases. In other words, the driver shakes the steering wheel less, which is clear from the low amplitude of EMG signals in Figure 15b. In addition, according to Figure 15b, the number of inhales and exhales in the fatigue state is reduced and becomes choppy, which indicates the driver’s fatigue. 

## 6. Conclusions

This study presents a new method for automatic driver fatigue detection based on EEG signals, which, in turn, are based on deep learning methods. In this study, a standard driving simulator was designed, and a benchmark database of 20 participants was registered. The proposed model is based on the combination of a GAN network and a convolutional graph. The proposed deep network consists of five convolutional graph layers along with a fully connected layer. The current proposed model can identify five different levels of fatigue and classify them in four application cases with an accuracy of over 90%. The present study has been compared with other methods and recent studies and has provided an acceptable performance. In future work, we plan to implement the proposed GCN model in real-time in the real world and measure its performance.

## Figures and Tables

**Table 1:** Summary of the performance of previous studies reviewed.

References	Method	Performance (%)
*Ren* et al. [12]	PCA-PSE	88
*Fouad* et al. [13]	Hand-Crafted Features-SVM	98
*Kumar* et al. [14]	ELM-SVM	98
*Lee* et al. [15]	PSD	95
*Ha* et al. [16]	Fuzzy delay index-SVM	93
*Abdubrani* et al. [17]	WT-CNN	98
*Sheykhivand* et al. [18]	CNN-LSTM	99
*Sheykhivand* et al. [19]	CS-CNN	87
*Peivandi* et al. [20]	CNN-typ 2 fuzzy	89
*Gao* et al. [21]	RNN	88
*Chen* et al. [22]	CapsNet-SACC	94.5

**Table 2:** Layers, weights, bias, and parameters in the proposed GCN model.

L	Weight	Bias	Parameters
First Layer	C_1_, 2920, 2920	2920	8,526,400 × C_1_ + 2920
BN	2920	2920	5840
Second Layer	C_2_, 2920, 1460	1460	4,263,200 × C_2_ + 1460
BN	1460	1460	2920
Third Layer	C_3_, 1460, 730	730	1,065,800 × C_3_ + 730
BN	730	730	1460
Fourth Layer	C_4_, 730, 365	365	266,450 × C_4_ + 365
BN	365	365	730
Fifth Layer	C_5_, 365, 180	180	65,700 × C_5_ + 180
BN	180	180	180
Flattening Layer	720, 4	4	2880

**Table 3:** Layers, weights, bias, and parameters in the proposed GAN-GCN model.

Parameters	Values Used	Optimal Value
Batch Size in GAN	8, 10	10
Optimizer in GAN	Adam, SGD,	Adam
Number of CNN Layers	3, 4, 5	4
Learning Rate in GAN	0.1, 0.01, 0.001	0.001
Number of Graph Conv Layers	2, 3, 4, 5, 6	5
Batch Size in GCN	8, 16	16
Learning Rate in GCN	0.1, 0.01, 0.001, 0.0001, 0.00001	0.0001
Dropout Rate	0.1, 0.2, 0.3	0.3
Weight of optimizer	$4\times 10^{-3},4\times 10^{-4},4\times 10^{-5},4\times 10^{-6}$	$4\times 10^{-4}$
Error function	MSE, Cross Entropy	Cross Entropy
Optimizer in GCN	Adam, SGD, Adadelta, Adamax	Adamax

**Table 4:** Scatter plot of samples for fully connected layer in GCN model.

Index	Accuracy (%)	Sensitivity (%)	Specificity (%)	Precision (%)	Kappa Coefficient
Case I	99.4	99.4	99.5	99.3	0.97
Case II	97.6	96.7	97.2	97.1	0.95
Case III	96.3	96.1	95.7	96.5	0.94
Case IV	91.7	93.4	92.4	90.1	0.89

## Data Availability

In this research, experimental data were not recorded.
